# Dynamic Model Validation and Simulation of Acetone–Toluene and Benzene–Toluene Systems for Industrial Volatile Organic Compound (VOC) Abatement

**DOI:** 10.1021/acs.iecr.4c00251

**Published:** 2024-04-15

**Authors:** Vasiliki E. Tzanakopoulou, Michael Pollitt, Daniel Castro-Rodriguez, Dimitrios I. Gerogiorgis

**Affiliations:** † Institute for Materials & Processes (IMP), School of Engineering, University of Edinburgh, Edinburgh EH9 3FB, U.K.; ‡ GlaxoSmithKline (GSK), Montrose, Angus DD10 8EA, Scotland, U.K.; § Haleon. No. 5 The Heights, Brooklands Business Park, KT13 0NY Weybridge, U.K.

## Abstract

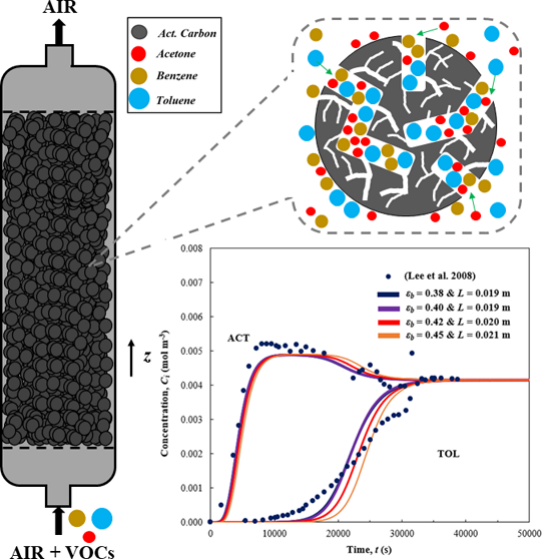

Environmental impact mitigation is one of the grand challenges for industries globally. Volatile organic compounds (VOCs) are solvents whose emissions are potentially toxic to human health and ecosystems yet indispensable for the manufacturing of life-saving medicine. Adsorption with activated carbon columns is an established countermeasure for end-of-pipe emission control, whose efficiency, however, is impeded by irregular bed saturation due to the complex nature of its inputs. This work presents the application of a validated nonisothermal adsorption model to examine multicomponent trace mixtures of acetone–toluene and benzene–toluene on activated carbon. Our results indicate preferential adsorption of toluene over both acetone and benzene for all concentrations examined, which is in agreement with experimental data. Moreover, moderate temperature variations and pressure drops are revealed. Finally, Glueckauf’s hodograph theory is employed for maximum outlet concentration prediction and compared with simulation results and experimental data, thus providing valuable insights into nonisothermal VOC abatement, which paves the way for industrial operation optimization.

## 1 Introduction and Motivation

The United Nations sustainable development goals (SDGs) provide a shared vision for now and the future, highlighting a responsible way forward for the global community. At the heart of this vision is tackling waste- and energy-intensive production patterns that are responsible for climate change and pollution (Goal 12). Leading this effort is the wider adoption of eco-friendly and innovative industrialization practices across all manufacturing sectors (Goal 9). Integrating technology in industrial settings not only benefits the environment but also increases productivity and resilience in crises.^1^ In fact, Industry 4.0 paves the way to a new manufacturing paradigm, focused on increased system predictability toward efficiency maximization and environmental impact and cost minimization.^2^

**Figure 1:**
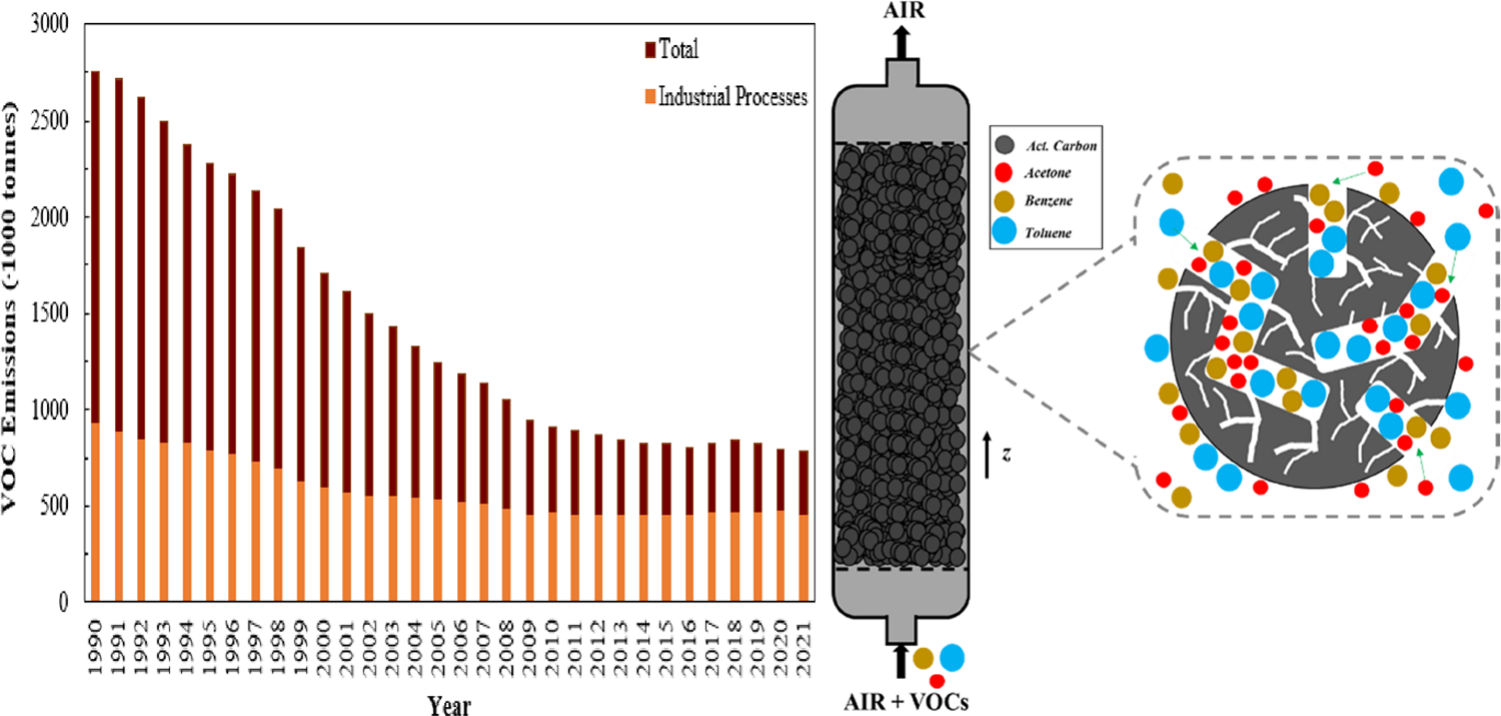
(a) VOC emissions in the UK (1990–2021).^3^ (b) VOC adsorption on an activated carbon bed.

Volatile organic compounds (VOCs) are solvents that are essential to primary pharmaceutical manufacturing. Their presence, ubiquitous in reactions and separations, results in a significant amount of vapor emissions from pharmaceutical production. Their diffuse nature has adverse effects in multiple sectors, including human health, the environment, and agriculture. Specifically, VOCs are responsible for ground ozone layer formation, which is known to not only cause oxidative damage in crops but also trigger inflammation and asthma in humans and contribute to the formation of particulate matter in the atmosphere.^3^ While decisive measures targeting VOC emissions have borne fruit over the past few decades (Figure 1a), there is still a heightened urgency to reach ambitious environmental protection targets by 2030 as industrial processes continue to contribute over 50% to the overall VOC emissions in the UK. To this end, optimization of existing VOC controlling infrastructure at source points is critical.

Adsorption with activated carbon is an established VOC emission control technology on an industrial level due to its easy installation and maintenance as well as energy efficiency. During adsorption (Figure 1b), a VOC-laden air stream, from process vents across the plant, is directed toward a fixed-bed activated carbon column. There, VOCs are selectively retained in the carbon pores via attraction forces, while the air stream passes through the bed and is released to the atmosphere, free from VOCs. Adsorption is often preferred due to its ability to filter large volumes of waste streams with a low concentration of pollutants. However, venting variable component and concentration waste streams to the activated carbon bed from process equipment all over the plant leads to suboptimal performance and irregular bed saturation.^4^

Despite the abundance of published adsorption studies, the number of papers directly addressing the adsorption of pharma-related VOCs is relatively small (Table 1). Notably, many published studies do not explicitly report parameters crucial for simulation purposes (e.g., bed length). This number becomes even smaller for studies under realistic industrial operating conditions and on equipment scales. One of the key characteristics of industrial waste streams in the pharma industry is the release of trace amounts of solvent vapors depending on the process stage and process unit, leading to multicomponent mixtures in need of abatement.

**Table 1:** Recent Studies Including Pharmaceutically Relevant VOC Adsorption on Activated Carbon (Adapted from Tzanakopoulou et al.^4^)

*N*_voc_	Multicomp.	Expt.	Sim.	VOC(s)	Adsorbent	Isotherm	*C*_0,max_ (ppm)	*L* (m)	Lit. Ref.
1	no	yes	yes	DCM	activ. carbon	Langmuir	8972		(6)
1	no	yes	yes	ethyl acetate	activ. carbon	Langmuir	200 876	6 × 10^–2^	(7)
1	no	yes	yes	*n*-hexane	activ. carbon	Langmuir, modified Langmuir 1–2, MSL, FH-VSM, Toth, Langmuir–Freundlich	100 000	1 × 10^–1^	(8)
2	yes	yes	yes	acetone, DCM	activ. carbon	Langmuir–Freundlich	8000	1 × 10^–1^	(9)
2	no	yes	yes	toluene xylene	activ. carbon fiber	Sips	10 000	1 × 10^–1^	(10)
2	no	yes	yes	DCM, 1,1,2-trichloro-l,2,2-trifluoroethane	activ. carbon	Langmuir, Sips, Freundlich	246 732	(1.5–2.5) × 10^–1^	(11)
2	no	yes	yes	TCM, carbon tetrachloride	activ. carbon	Langmuir, Freundlich, DRK, BET	1250	3 × 10^–1^	(12)
2	no	yes	no	acetone, benzene	activ. carbon fiber cloth	Dubinin-Ashtakhov	19 600		(13)
2	no	yes	no	toluene, ethyl acetate	activ. carbon	Langmuir, Freundlich, Sips, Toth, Redlich-Peterson	1666	2.2 × 10^–1^	(14)
2	no	Yes	No	toluene, *n*-hexane	activ. carbon	Langmuir	910 000		(15)
2	no	yes	no	toluene, chlorobenzene	activ. carbon		14		(16)
2	no	yes	no	benzene, toluene	activ. carbon	IAST, Toth	200	(6–10) × 10^–3^	(17)
3	yes	yes	no	acetaldehyde, acetone, ethyl acetate	activ. carbon	Yoon-Nelson	208	6 × 10^–1^	(18)
3	yes	yes	no	toluene, MEK, MiBK	activ. carbon	Freundlich, Myers	250 000		(19)
3	yes	yes	yes	benzene, toluene, *p*-xylene	activ. carbon	Langmuir	3175	1 × 10^–1^	(20)
3	no	yes	yes	TCM, benzene, carbon tetrachloride	activ. carbon	Langmuir	6966	9 × 10^–3^	(21)
3	no	yes	yes	acetone, ethyl formate, DCM	activ. carbon	Langmuir	28 322	2 × 10^–1^	(22)
3	no	yes	yes	acetone, toluene, 1,2-dichloroethane	activ. carbon	Langmuir	41 415	2 × 10^–1^	(23)
3	no	yes	no	benzene, toluene, *p*-xylene	activ. carbon				(24)
3	no	yes	no	MCM, DCM, TCM	activ. carbon	Langmuir	4000	(4–11) × 10^–2^	(25)
3	no	yes	no	acetone, TCM, acetonitrile	activ. carbon, activ. carbon fiber, sludge	Langmuir, Freundlich	7800		(26)
3	no	yes	no	toluene, methanol, acetone	activ. carbon fiber		1263		(27)
3	no	yes	no	DCM, TCM, carbon tetrachloride	activ. carbon	Langmuir, Freundlich, Temkin	5761		(28)
3	no	yes	no	ethyl acetate, acetone, ethanol	activ. carbon	Langmuir	197 386		(29)
4	yes	yes	yes	acetone, ethanol, cyclohexane, heptane	activ. carbon	Langmuir	6000	1 × 10^–1^	(30)
4	yes	yes	no	acetone, MEK, benzene, toluene	activ. carbon		160		(5)
4	yes	yes	no	acetone, ethanol, ethyl acetate, *N*-propyl acetate	activ. carbon fiber				(31)
4	yes	yes	no	benzene, toluene, styrene, xylene	activ. carbon	Langmuir, Dubinin–Radushkevich, Freundlich, Elovich	100	2 × 10^–2^	(32)
4	no	yes	yes	acetone, benzene, TMB, toluene	activ. carbon	Langmuir	1000	1.5 × 10^–1^	(33)
4	no	yes	no	DCM, TCM, benzene, carbon tetrachloride	activ. carbon	Langmuir	400		(34)
5	yes	yes	no	acetaldehyde, formaldehyde, 2-propenal, 1,3-butadiene, benzene	activ. carbon		1400		(35)
6	no	yes	yes	acetone, DCM, ethyl acetate, isopropanol, MEK, toluene	activ. carbon fiber	Langmuir	1050	(1.64–5) × 10^–3^	(36)
7	no	yes	yes	acetone, 1,2-dichloroethane, ethyl acetate, ethyl alcohol, methylethyldioxolane, MEK, toluene	activ. carbon	Langmuir	27 300	2 × 10^–1^	(37)
8	yes	yes	yes	1,2,4-trimethylbenzene, 2,2-dimethylpropylbenzene, indane, decane, heptane, 2-butoxyethanol, 2-heptanone, *n*-butyl acetate, *n*-butanol	activ. carbon	Langmuir	250	6.5 × 10^–2^	(38)
8	yes	yes	no	1-butanol, *n*-butyl acetate, 2-heptanone, 2-butoxyethanol *n*-decane, 1,2,4-trimethylbenzene, indane, and 2,2-dimethylpropylbenzene	activ. carbon		500		(39)

This paper constitutes an effort to address a critical need to comprehend multicomponent pharmaceutical VOC adsorption for industrial operations, even when source data are limited. Specifically, it demonstrates the application of a validated, dynamic, nonisothermal, VOC adsorption model to highlight the breakthrough characteristics of two binary VOC mixtures: acetone–toluene and benzene–toluene, in three pairs of concentrations (160–40, 100–100, and 40–160 ppm) and validates them against published experimental data.^5^ Moreover, Glueckauf’s hodograph theory is employed to predict the maximum column outlet concentration of the weakly adsorbing component and is compared against gPROMS simulation results and published experimental data in an effort to improve modeling accuracy and pave the way for process optimization on the industrial scale.

## 2 Computational Details: Dynamic Model Development

The validated fixed bed, multicomponent, nonisothermal adsorption model,^4^ considering mass and energy balances in the axial dimension, is employed to describe binary VOC mixture adsorption under industrially relevant conditions in this work. The mass transfer between the gas phase and the solid particles, as well as heat transfer from inside the column to the environment, are described using lumped equations. The mathematical model used in this work relies on the following assumptions:

1. Radial concentration and temperature gradients are negligible.^40^
2. The gas phase and adsorbent particles are in thermal equilibrium.^41^
3. Wall temperature is constant and equal to the ambient temperature.^41^
4. The ideal gas law applies, and carrier gas adsorption is negligible.^41^
5. Initially (*t* = 0 s), the column only contains carrier gas.^40^
6. Equilibrium obeys the Extended Langmuir model for mixtures.^33^

Considering all of the assumptions, the overall and component mass balances are given as follows (*i*: component)
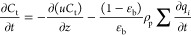
1
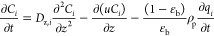
2where *C*_t_ is the total gas-phase VOC concentration, *C_i_* is the component *i* gas-phase VOC concentration, *D*_z,*i*_ is the axial dispersion coefficient of component *i*, *u* is the interstitial velocity, ε_b_ is the bulk bed porosity, ρ_p_ is the particle density, and *q* the adsorbed phase VOC concentration.

The axial dispersion coefficient of component *i* is calculated by^33^
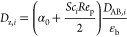
3where Sc_*i*_ is the Schmidt number of *i*, Re_p_ is the Reynolds number (adsorbent particle), *D*_AB,*i*_ is the molecular diffusivity, and α_0_ is the empirical mass diffusion correction factor.

The molecular diffusivity of component *i* is estimated by
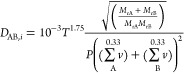
4where *∑ν* is the atomic diffusion volume (A: VOC, B: carrier), *T* is the temperature, *P* is the pressure, and *M*_r_ is the molecular weight.

Solid-phase adsorption is modeled by the established linear driving force (LDF) model, which is characterized as simple, analytic, and physically consistent,^42^ considering a lumped overall mass transfer coefficient^33,38^
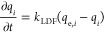
5where *k*_LDF,*i*_ is the LDF mass transfer coefficient and *q*_e,*i*_ is the inlet PT adsorbent equilibrium capacity.

The LDF mass transfer coefficient is estimated by^33,38^
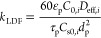
6where ε_p_ is the particle porosity, *C*_0,*i*_ is the inlet concentration of *i*, *D*_eff,*i*_ is the effective diffusivity of *i*, τ_p_ is the particle tortuosity, *C*_s0,*i*_ is the adsorbed phase concentration at equilibrium with *C*_0,*i*_, and *d*_p_ is the particle diameter.

The particle density is given by^33,38^
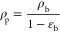
7where ρ_b_ is the bed density and ε_b_ is the bed porosity.

The bed porosity is calculated by^33,38^
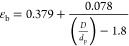
8where *D* is the column’s internal diameter and *d*_p_ is the particle diameter.

The particle porosity is calculated by^33,38^
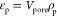
9where *V*_pore_ is the adsorbent pore volume.

The particle tortuosity is given by^33,38^
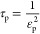
10where ε_p_ is the particle porosity.

The adsorbed phase concentration at equilibrium with *C*_0,*i*_ is given by^33,38^
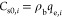
11where ρ_b_ is the bed porosity and *q*_e,*i*_ is the inlet PT adsorbent equilibrium capacity.

The Knudsen diffusivity is estimated by^40^
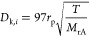
12where *D*_k,*i*_ is the Knudsen diffusivity, *r*_p_ is the average pore radius, and *T* and *M*_rA_ are the temperature and VOC molecular weight, respectively.

The effective diffusivity is given by^33,38^
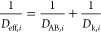
13

The Bosanquet formula of eq 13, thus eq 6, is verified for the estimation of the effective diffusivity (*D*_eff,*i*_).^43^

Adsorption equilibrium is assumed to obey the Extended Langmuir Model, which is described as follows
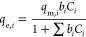
14
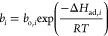
15where *q*_e,*i*_ is the equilibrium adsorption capacity of *i*, *q*_m,*i*_ is the maximum adsorption capacity of *i*, *b*_*i*_ is the Langmuir affinity coefficient, *b*_o,*i*_ is the pre-exponential Langmuir affinity coefficient constant, and Δ*H*_ad,*i*_ is the heat of adsorption.

The energy balance for the fluid and solid phases, as well as the parameter main equations, are as follows^40,41,44^
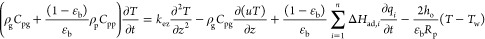
16where *T*_w_ is the wall temperature, ρ_g_ is the gas density, *C*_pg_ is the specific heat capacity of the gas, *C*_pp_ is the specific heat capacity of the particle, *k*_ez_ is the effective axial thermal conductivity, *R*_p_ is the particle radius, *T* is the temperature, and *h*_o_ is the overall heat transfer coefficient.

The effective thermal conductivity is calculated by^44^
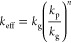
17
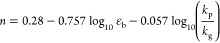
18where *k*_eff_ is the effective thermal conductivity, *k*_g_ is the gas thermal conductivity, *k*_p_ is the particle thermal conductivity, and *n* is the Krupicka equation parameter.

The effective axial thermal conductivity is calculated by^44^
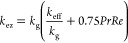
19

The overall heat transfer coefficient is given by^40^
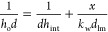
20where *h*_int_ is the internal heat transfer coefficient, *k*_w_ is the wall thermal conductivity, *x* is the wall thickness, and *d*_lm_ is the mean logarithmic column diameter.

The internal heat transfer coefficient is given by^40^
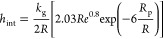
21where *R* is the internal column radius.

The pressure drop along the column is calculated using Ergun’s equation^40,41^
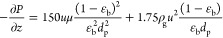
22where μ is the gas viscosity and *P* is the pressure.

The system boundary conditions at the column inlet (*z* = 0) can be written as follows
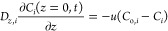
23
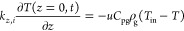
24
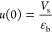
25

The boundary conditions at the column outlet (*z* = *L*) are
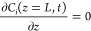
26
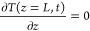
27
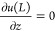
28

The initial conditions at *t* = 0 for 0 *≤ z ≤ L*
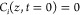
29
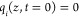
30
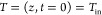
31

### 2.1 Dynamic Model Parameters for Case Studies

The developed model was employed to investigate the adsorption characteristics of binary VOC mixtures (with air as the carrier gas) as previously published.^5^ The model examines specifically the mixtures of acetone–toluene and benzene–toluene in an initially clean, coconut-based activated carbon fixed bed at three different concentration pairs each. The calculation of Langmuir Isotherm parameters was beyond the scope of the original publication^5^—hence, in this work, the necessary Langmuir Isotherm parameter values are taken from the literature^33^ for all components, as shown in Table 2.

**Table 2:** Langmuir Isotherm Parameters Used in This Study

binary	*q*_m,*i*_ (mol kg^–1^)	*b*_0_ (m^3^ mol^–1^)	Δ**H**_ad,*i*_ (J mol^–1^)	lit. ref.
ACT–TOL	7.06	1.96 × 10^–8^	51 125	(33)
	4.56	1.27 × 10^–8^	59 722	(33)
BEN–TOL	5.38	1.13 × 10^–8^	56 027	(33)
	4.56	1.27 × 10^–8^	59 722	(33)

In order to perform the model validation for the published experimental data^5^ on coconut-based activated carbon and given that the bed porosity and bed density were not reported, an initial estimation was performed by assuming the value of 528.61 kg m^–3^ based on commercial coconut-based activated carbon specifications^45^ over a range of typical bed porosity fractions (ε_b_ = 0.38–0.45). The computational determination of column length is based on the activated carbon mass previously reported,^5^ by solving the following equation system (eqs 32 and 33)
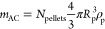
32
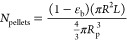
33

The results of the estimated column length based on different bed porosities and a coconut-based activated carbon density are presented in Table 3.

**Table 3:** Column Structural Properties Calculation Results

*R*_p_ (m)	*R* (m)	ε_b_	ρ_b_ (kg m^–3^)	ρ_p_ (kg m^–3^)	*m*_ac_ (kg)	*L* (m)
0.001	0.008	0.38	528.61	852.60	0.002	0.019
0.001	0.008	0.40	528.61	881.02	0.002	0.019
0.001	0.008	0.42	528.61	911.40	0.002	0.020
0.001	0.008	0.45	528.61	961.11	0.002	0.021

The set of partial differential equations (PDEs) is solved using second-order orthogonal collocation on finite elements with 50 discretization points in the gPROMS Process 2.0.0 software suite. For all cases considered, the DASolver SRADAU is employed, which uses a variable time step with a fully implicit Runge–Kutta method. The viscosities are computed from Wilke’s equation, while densities are determined through pure component data via mixing rules.^46^ Each of the six binary mixtures is solved for four different cases corresponding to four different bed porosities (ε_b_ = 0.38, 0.40, 0.42, and 0.45) and their corresponding bed lengths as resulting from calculations in Table 3. The main simulation parameters for all acetone–toluene (ACT–TOL) cases examined in this study are listed in Table 4.

**Table 4:** Model Parameter Values for the Acetone–Toluene Binary Simulations

System	*C*_0,*i*_ (ppm)	*D*_*z*,*i*_ (m^2^ s^–1^)	*T*_in_ (K)	*L* (m)	*V*_s_ (m s^–1^)	ε_b_	*k*_LDF_ (s^–1^)	Figure
ACT–TOL	160	1.43 × 10^–3^	293.15	0.019	0.332	0.38	9.91 × 10^–6^	2–5
	40	1.30 × 10^–3^					5.52 × 10^–7^	
ACT–TOL	160	1.35 × 10^–3^	293.15	0.019	0.332	0.40	1.09 × 10^–5^	2–5
	40	1.23 × 10^–3^					6.09 × 10^–7^	
ACT–TOL	160	1.29 × 10^–3^	293.15	0.020	0.332	0.42	1.21 × 10^–5^	2–5
	40	1.17 × 10^–3^					6.74 × 10^–7^	
ACT–TOL	160	1.20 × 10^–3^	293.15	0.021	0.332	0.45	1.42 × 10^–5^	2–5
	40	1.09 × 10^–3^					7.91 × 10^–7^	
ACT–TOL	100	1.43 × 10^–3^	293.15	0.019	0.332	0.38	1.62 × 10^–5^	2–5
	100	1.30 × 10^–3^					9.02 × 10^–7^	
ACT–TOL	100	1.35 × 10^–3^	293.15	0.019	0.332	0.40	1.79 × 10^–5^	2–5
	100	1.23 × 10^–3^					9.95 × 10^–7^	
ACT–TOL	100	1.29 × 10^–3^	293.15	0.020	0.332	0.42	1.98 × 10^–5^	2–5
	100	1.17 × 10^–3^					1.10 × 10^–6^	
ACT–TOL	100	1.20 × 10^–3^	293.15	0.021	0.332	0.45	2.32 × 10^–5^	2–5
	100	1.09 × 10^–3^					1.29 × 10^–6^	
ACT–TOL	40	1.43 × 10^–3^	293.15	0.019	0.332	0.38	2.25 × 10^–5^	2–5
	160	1.30 × 10^–3^					1.25 × 10^–6^	
ACT–TOL	40	1.35 × 10^–3^	293.15	0.019	0.332	0.40	2.48 × 10^–5^	2–5
	160	1.23 × 10^–3^					1.38 × 10^–6^	
ACT–TOL	40	1.29 × 10^–3^	293.15	0.020	0.332	0.42	2.74 × 10^–5^	2–5
	160	1.17 × 10^–3^					1.53 × 10^–6^	
ACT–TOL	40	1.20 × 10^–3^	293.15	0.021	0.332	0.45	3.22 × 10^–5^	2–5
	160	1.09 × 10^–3^					1.79 × 10^–6^	

Table 5 introduces the main simulation parameters for all benzene–toluene (BEN–TOL) mixtures examined in this study.

**Table 5:** Main Parameter Values for the Benzene–Toluene Binary Simulations

System	*C*_0,*i*_ (ppm)	*D*_*z*,*i*_ (m^2^ s^–1^)	*T*_in_ (K)	*L* (m)	*V*_s_ (m s^–1^)	ε_b_	*k*_LDF_ (s^–1^)	Figure
BEN–TOL	160	1.34 × 10^–3^	293.15	0.019	0.332	0.38	3.29 × 10^–6^	2–5
	40	1.30 × 10^–3^					6.99 × 10^–7^	
BEN–TOL	160	1.27 × 10^–3^	293.15	0.019	0.332	0.40	3.63 × 10^–6^	2–5
	40	1.23 × 10^–3^					7.71 × 10^–7^	
BEN–TOL	160	1.21 × 10^–3^	293.15	0.020	0.332	0.42	4.02 × 10^–6^	2–5
	40	1.17 × 10^–3^					8.53 × 10^–7^	
BEN–TOL	160	1.13 × 10^–3^	293.15	0.021	0.332	0.45	4.72 × 10^–6^	2–5
	40	1.09 × 10^–3^					1.00 × 10^–6^	
BEN–TOL	100	1.34 × 10^–3^	293.15	0.019	0.332	0.38	4.68 × 10^–6^	2–5
	100	1.30 × 10^–3^					9.93 × 10^–7^	
BEN–TOL	100	1.27 × 10^–3^	293.15	0.019	0.332	0.40	5.17 × 10^–6^	2–5
	100	1.23 × 10^–3^					1.10 × 10^–6^	
BEN–TOL	100	1.21 × 10^–3^	293.15	0.020	0.332	0.42	5.72 × 10^–6^	2–5
	100	1.17 × 10^–3^					1.21 × 10^–6^	
BEN–TOL	100	1.13 × 10^–3^	293.15	0.021	0.332	0.45	6.71 × 10^–6^	2–5
	100	1.09 × 10^–3^					1.42 × 10^–6^	
BEN–TOL	40	1.34 × 10^–3^	293.15	0.019	0.332	0.38	6.07 × 10^–6^	2–5
	160	1.30 × 10^–3^					1.29 × 10^–6^	
BEN–TOL	40	1.27 × 10^–3^	293.15	0.019	0.332	0.40	6.70 × 10^–6^	2–5
	160	1.23 × 10^–3^					1.42 × 10^–6^	
BEN–TOL	40	1.21 × 10^–3^	293.15	0.020	0.332	0.42	7.42 × 10^–6^	2–5
	160	1.17 × 10^–3^					1.57 × 10^–6^	
BEN–TOL	40	1.13 × 10^–3^	293.15	0.021	0.332	0.45	8.70 × 10^–6^	2–5
	160	1.09 × 10^–3^					1.84 × 10^–6^	

Table 6 summarizes the main structural (column and adsorbent) and thermal parameter values of the acetone–toluene (ACT–TOL) simulations. Values for *C*_pp_ and *k*_w_ are taken from the literature^33^ for coconut-based activated carbon.

**Table 6:** Main Structural and Thermal Parameter Values of the Systems

System	*C*_0,*i*_ (ppm)	*D* (m)	*T*_w_ (K)	ε_p_	*d*_p_ (m)	*C*_pp_ (J kg^–1^ K^–1^)	*C*_pg_ (J kg^–1^ K^–1^)	*k*_ez_ (W m^–1^ K^–1^)	*h*_o_ (W m^–2^ K^–1^)	*h*_int_ (W m^–2^ K^–1^)	*k*_w_ (W m^–1^ K^–1^)	*x* (m)	Figure
ACT–TOL	160	0.016	293.15	0.52	0.002	706.7	1013	0.66	31.54	31.60	14.2	0.001	2–5
	40												
ACT–TOL	160	0.016	293.15	0.54	0.002	706.7	1013	0.66	31.54	31.60	14.2	0.001	2–5
	40												
ACT–TOL	160	0.016	293.15	0.56	0.002	706.7	1013	0.66	31.54	31.60	14.2	0.001	2–5
	40												
ACT–TOL	160	0.016	293.15	0.59	0.002	706.7	1013	0.66	31.54	31.60	14.2	0.001	2–5
	40												
ACT–TOL	100	0.016	293.15	0.52	0.002	706.7	1013	0.66	31.56	31.63	14.2	0.001	2–5
	100												
ACT–TOL	100	0.016	293.15	0.54	0.002	706.7	1013	0.66	31.56	31.63	14.2	0.001	2–5
	100												
ACT–TOL	100	0.016	293.15	0.56	0.002	706.7	1013	0.66	31.56	31.63	14.2	0.001	2–5
	100												
ACT–TOL	100	0.016	293.15	0.59	0.002	706.7	1013	0.66	31.56	31.63	14.2	0.001	2–5
	100												
ACT–TOL	40	0.016	293.15	0.52	0.002	706.7	1013	0.66	31.58	31.65	14.2	0.001	2–5
	160												
ACT–TOL	40	0.016	293.15	0.54	0.002	706.7	1013	0.66	31.58	31.65	14.2	0.001	2–5
	160												
ACT–TOL	40	0.016	293.15	0.56	0.002	706.7	1013	0.66	31.58	31.65	14.2	0.001	2–5
	160												
ACT–TOL	40	0.016	293.15	0.59	0.002	706.7	1013	0.66	31.58	31.65	14.2	0.001	2–5
	160												

Table 7 introduces the thermal and column structural properties of the benzene–toluene mixture cases. Values for *C*_pp_ and *k*_w_ are taken from the literature^33^ for coconut-based activated carbon.

**Table 7:** Thermal and Structural Properties for Benzene–Toluene Binary Simulations

System	*C*_0,*i*_ (ppm)	*D* (m)	*T*_w_ (K)	ε_p_	*d*_p_ (m)	*C*_pp_ (J kg^–1^ K^–1^)	*C*_pg_ (J kg^–1^ K^–1^)	*k*_ez_ (W m^–1^ K^–1^)	*h*_o_ (W m^–2^ K^–1^)	*h*_int_ (W m^–2^ K^–1^)	*k*_w_ (W m^–1^ K^–1^)	*x* (m)	Figure
BEN–TOL	160	0.016	293.15	0.52	0.002	706.7	1013	0.66	31.56	31.63	14.2	0.001	2–5
	40												
BEN–TOL	160	0.016	293.15	0.54	0.002	706.7	1013	0.66	31.56	31.63	14.2	0.001	2–5
	40												
BEN–TOL	160	0.016	293.15	0.56	0.002	706.7	1013	0.66	31.56	31.63	14.2	0.001	2–5
	40												
BEN–TOL	160	0.016	293.15	0.59	0.002	706.7	1013	0.66	31.56	31.63	14.2	0.001	2–5
	40												
BEN–TOL	100	0.016	293.15	0.52	0.002	706.7	1013	0.66	31.56	31.63	14.2	0.001	2–5
	100												
BEN–TOL	100	0.016	293.15	0.54	0.002	706.7	1013	0.66	31.56	31.63	14.2	0.001	2–5
	100												
BEN–TOL	100	0.016	293.15	0.56	0.002	706.7	1013	0.66	31.56	31.63	14.2	0.001	2–5
	100												
BEN–TOL	100	0.016	293.15	0.59	0.002	706.7	1013	0.66	31.56	31.63	14.2	0.001	2–5
	100												
BEN–TOL	40	0.016	293.15	0.52	0.002	706.7	1013	0.66	31.60	31.66	14.2	0.001	2–5
	160												
BEN–TOL	40	0.016	293.15	0.54	0.002	706.7	1013	0.66	31.60	31.66	14.2	0.001	2–5
	160												
BEN–TOL	40	0.016	293.15	0.56	0.002	706.7	1013	0.66	31.60	31.66	14.2	0.001	2–5
	160												
BEN–TOL	40	0.016	293.15	0.59	0.002	706.7	1013	0.66	31.60	31.66	14.2	0.001	2–5
	160												

### 2.2 Results and Discussion: Dynamic Simulations (gPROMS)

The developed model was validated against published experimental data^5^ for the adsorption of trace binary mixtures of acetone–toluene (ACT–TOL) and benzene–toluene (BEN–TOL) on coconut-based activated carbon for three concentration pairs each (160–40, 100–100, and 40–160 ppm) using air as the nonadsorbing carrier gas. Each of the six binaries was simulated for four different bed porosity fractions (ε_b_ = 0.38, 0.40, 0.42, and 0.45) and their corresponding bed lengths as calculated by eqs 32 and 33 in Table 3.

**Figure 2:**
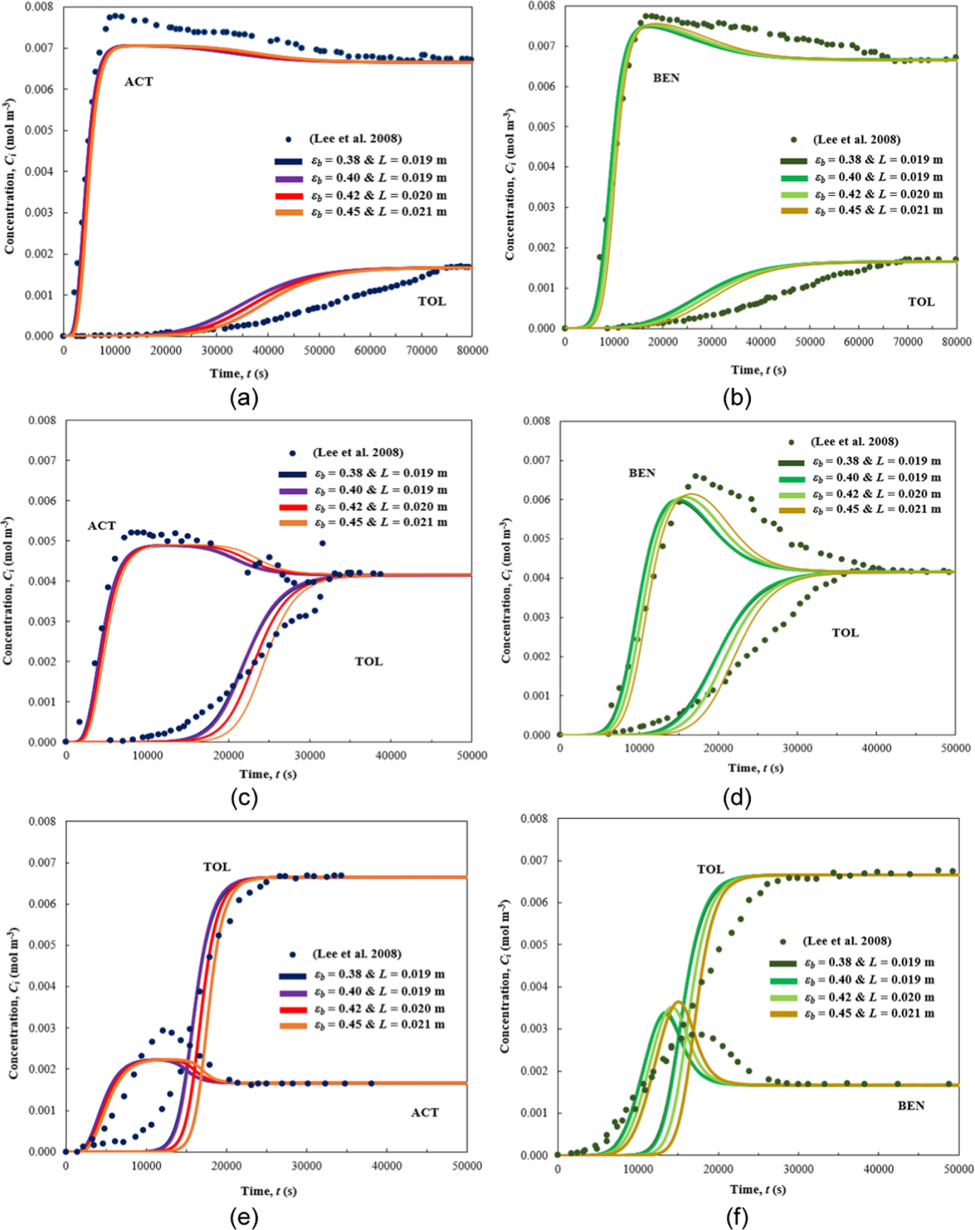
Breakthrough curves from binary mixture dynamic simulations: (a, b) 160–40 ppm, (c, d) 100–100 ppm, and (e–f) 40–160 ppm.

**Figure 3:**
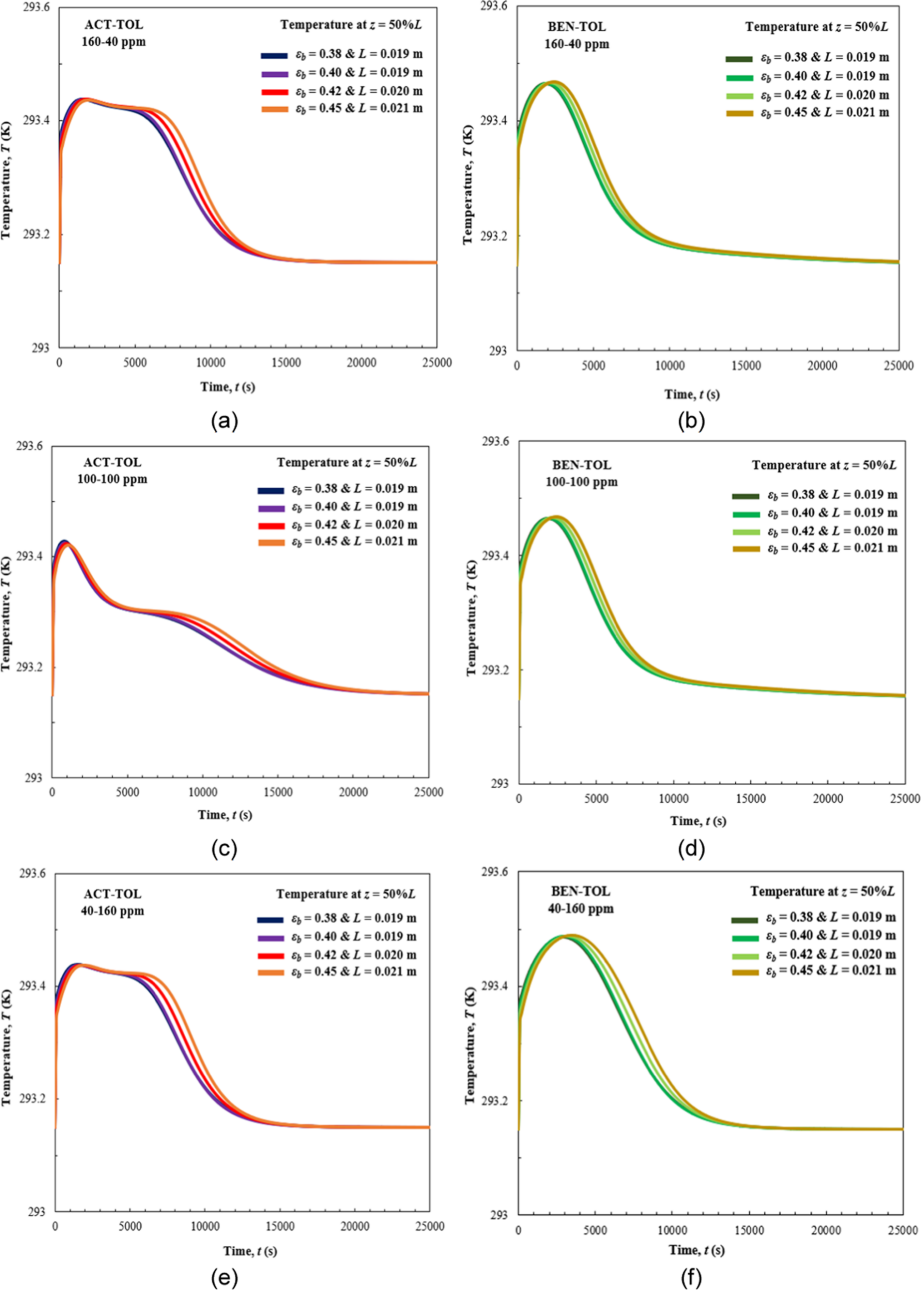
Temperature variation at the midpoint (*z* = 0.5 L) from binary mixture dynamic simulations. (a, b) 160–40 ppm, (c, d) 100–100 ppm, and (e–f) 40–160 ppm.

**Figure 4:**
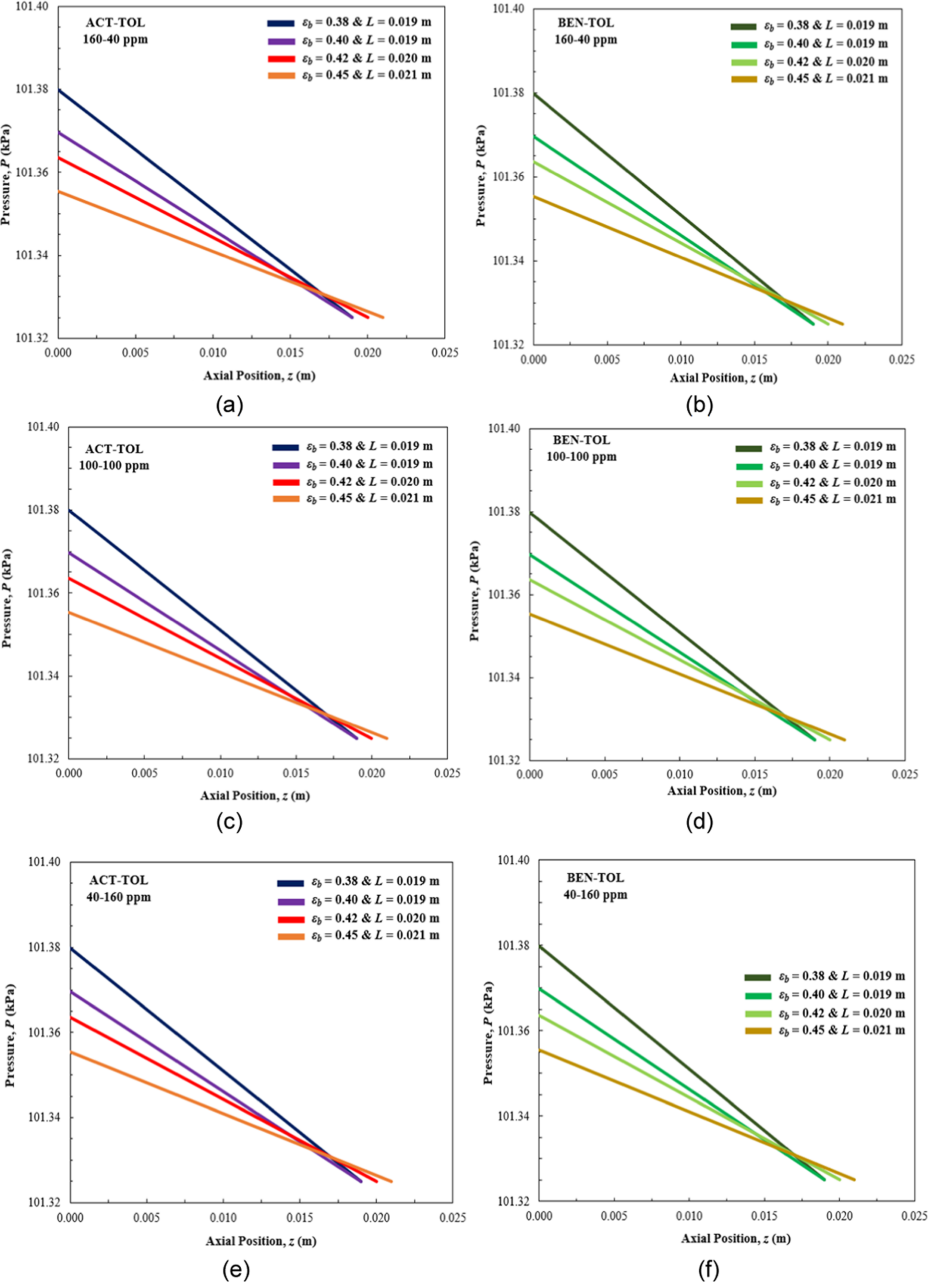
Pressure drop profile results from binary mixture dynamic simulations: (a, b) 160–40 ppm, (c, d) 100–100 ppm, and (e–f) 40–160 ppm.

Breakthrough curve (concentration at the column exit vs time) plots of the acetone–toluene and benzene–toluene binary mixtures are presented in Figure 2, while Figures 3 and 4 introduce the simulation results for temperature evolution in the middle of the adsorption columns and pressure drops, respectively. Tables 8 and 9 show key breakthrough time metrics. Specifically, the breakthrough onset time (*t*_5%_) is estimated as the time needed for the outlet concentration to reach 5% of the final concentration. Breakthrough completion time is here regarded as the time needed for the outlet concentration to reach 95% of the final concentration for the strongly adsorbing component (*t*_95%_) and 105% of the final concentration for the weakly adsorbing component (*t*_105%_). Lastly, the breakthrough duration (*t*_drt_) is the difference between breakthrough completion and onset times.

**Table 8:** Key Simulation Results and Time Metrics for the Acetone–Toluene Mixtures

Component	ε_b_	*C*_0_ (ppm)	*t*_5%_ (s)	*t*_105%_/*t*_95%_ (s)	*t*_drt_ (s)	*T*_max_ (K)	Δ*P* (Pa)	Figure 2–4
ACT	0.38	160	2042	9515	7472	293.4275	54.86	a
TOL		40	20 413	55 739	35 326			
ACT	0.40	160	2084	9465	7381	293.4266	44.71	
TOL		40	20 930	55 099	34 169			
ACT	0.42	160	2282	9788	7506	293.4260	38.59	
TOL		40	23 060	56 675	33 615			
ACT	0.45	160	2507	10 057	7550	293.4252	30.38	
TOL		40						
ACT	0.38	100	2142	6748	4606	293.4279	54.86	c
TOL		100	16 353	29 638	13 284			
ACT	0.40	100	2178	6748	4571	293.4254	44.71	
TOL		100	16 623	29 297	12 674			
ACT	0.42	100	2371	7068	4697	293.4235	38.59	
TOL		100	18 033	30 197	12 164			
ACT	0.45	100	2592	7383	4791	293.4211	30.38	
TOL		100	19 555	30 945	11 390			
ACT	0.38	40	2190	6021	3831	293.4386	54.86	e
TOL		160	13 055	19 984	6929			
ACT	0.40	40	2219	6036	3817	293.4374	44.71	
TOL		160	13 216	19 766	6550			
ACT	0.42	40	2413	6360	3947	293.4372	38.59	
TOL		160	14 217	20 424	6207			
ACT	0.45	40	2623	6683	4060	293.4369	30.38	
TOL		160	15 274	21 010	5735			

**Table 9:** Key Simulation Results and Time Metrics for the Benzene–Toluene Mixtures

Component	ε_b_	*C* (ppm)	*t*_5%_ (s)	*t*_105%_/*t*_95%_ (s)	*t*_drt_ (s)	*T*_max_ (K)	Δ*P* (Pa)	Figures 2–4
BEN	0.38	160	5658	12 869	7211	293.4645	54.86	b
TOL		40	14 341	47 117	32 775			
BEN	0.40	160	5765	12 782	7017	293.4651	44.71	
TOL		40	14 706	46 619	31 913			
BEN	0.42	160	6304	13 250	6946	293.4666	38.59	
TOL		40	16 209	47 992	31 783			
BEN	0.45	160	6893	13 687	6794	293.4682	30.38	
TOL		40	17 923	49 145	31 222			
BEN	0.38	100	5898	10 909	5011	293.4720	54.86	d
TOL		100	13 339	28 747	15 409			
BEN	0.40	100	5997	10 920	4923	293.4724	44.71	
TOL		100	13 591	28 440	14 849			
BEN	0.42	100	6537	11 479	4942	293.4737	38.59	
TOL		100	14 776	29 270	14 495			
BEN	0.45	100	7125	12 052	4926	293.4750	30.38	
TOL		100	16 099	30 034	13 935			
BEN	0.38	40	6056	10 272	4216	293.4863	54.86	f
TOL		160	12 126	20 077	7951			
BEN	0.40	40	6149	10 315	4166	293.4869	44.71	
TOL		160	12 280	19 859	7578			
BEN	0.42	40	6700	10 929	4229	293.4880	38.59	
TOL		160	13 222	20 538	7316			
BEN	0.45	40	7295	11 564	4270	293.4888	30.38	
TOL		160	14 228	21 111	6882			

Figure 2a presents the mixture of acetone–toluene at inlet concentrations of 160–40 ppm, respectively. Our model accurately captures the breakthrough onset and final concentrations for both components. For acetone, the slope of the breakthrough curve is successfully captured, whereas a slight mismatch is observed for toluene’s after breakthrough onset. The predicted maximum overshoot concentration observed at the column outlet for acetone is slightly underpredicted by the model compared to experimental data. Specifically, the difference between the experimental maximum outlet concentration for acetone and the simulation results is below 9.5% for all four potential porosities and corresponding bed lengths. For increasing bed porosity and thus column lengths, the breakthrough onset comes later for both components. For ε_b_ = 0.38, the breakthrough onset time for acetone comes at *t*_5%_ = 2042 s, while for toluene, it comes at *t*_5%_ = 20 413 s. Breakthrough completion for acetone takes place at *t*_105%_ = 9515 s and for toluene, at *t*_95%_ = 55 739 s.

The results for the mixture of acetone–toluene in 100 ppm of inlet concentration each can be found in Figure 2c. Good agreement is observed for both acetone and toluene, while a steeper slope for the sigmoidal concentration curve of toluene is predicted compared to experimental data. The maximum acetone concentration predicted at column exit is within a remarkable 6% relative error compared to experimental results for all four porosities and corresponding bed lengths. With increasing bed porosity, a longer column length is calculated (*L* = 0.019–0.021 m), and thus the breakthrough onset metrics for both components increase with increasing bed porosity. Comparing the ε_b_ = 0.38 case of 100–100 and 160–40 ppm, it is noted that the breakthrough onset time for acetone increases for the 100 ppm inlet concentration by ≈5%, while for toluene, it decreases by ≈20%. Breakthrough completion times for the same set of comparisons decreased by 29% for acetone and 47% for toluene. Thus, the overall duration for acetone decreased by 39% and for toluene by 63% for the ε_b_ = 0.38 case at 100–100 ppm compared to that at 160–40 ppm.

The results of the 40–160 ppm pair of concentrations for the acetone/toluene mixture are found in Figure 2e. In this scenario, the model accurately predicts the breakthrough onset time of acetone, while for toluene, the model curve is steeper than that of the experimental. The higher acetone concentration observed at the column outlet is predicted with a relative error below 25% for all bed porosity and corresponding bed lengths examined in this work. Comparison between the 40–160 and 100–100 ppm concentration pair for ε_b_ = 0.38 shows the breakthrough onset time taking place ≈2% later for acetone and ≈20% earlier for toluene at 40–160 ppm concentrations. Breakthrough completion between the same cases happens ≈11% earlier for acetone and ≈33% earlier for toluene at the 40–160 ppm compared to the 100–100 ppm concentrations. Breakthrough duration, hence, is shorter by ≈17% for acetone and ≈48% for toluene at 40–160 ppm compared to 100–100 ppm.

Figure 2b presents the results for the benzene–toluene mixture at an inlet concentration of 160–40 ppm, respectively. The model predicts in excellent agreement the breakthrough onset and curve slope for benzene, even capturing the maximum outlet concentration within ≈3% relative error. For toluene, while the breakthrough onset and final concentration are accurately predicted, there is a small mismatch with the curve’s slope. The breakthrough onset for benzene takes place at *t*_5%_*=* 5658 s for ε_b_ = 0.38 with a duration of *t*_drt_ = 7211 s. For toluene, the breakthrough onset starts later, at *t*_5%_*=* 14 341 s and lasts longer than acetone, with a duration of 32 775 s. For increasing bed porosity and thus column lengths, the breakthrough onset comes later for both components.

Figure 2d shows the breakthrough curve results for the binary mixture of benzene–toluene at 100–100 ppm inlet concentrations, respectively. The model accurately captures the benzene breakthrough onset and approaches the maximum outlet concentration with a relative error below 10%. Despite a small mismatch observed for the toluene curve slope and by association for the toluene breakthrough onset, the final toluene outlet concentration is accurately captured. Comparing the ε_b_ = 0.38 case of 100–100 ppm with 160–40 ppm shows that the breakthrough onset of benzene is ≈4% later and toluene’s ≈7% earlier than in 160–40 ppm. Breakthrough completion is ≈15% earlier for benzene and ≈39% earlier for toluene at the 100–100 ppm inlet concentration case compared to the 160–40 ppm binary for ε_b_ = 0.38. Finally, the breakthrough duration for benzene is ≈31% shorter, and for toluene ≈53% shorter in the 100–100 ppm inlet concentration case, compared to the 160–40 ppm case.

Figure 2f presents the breakthrough curves for the 40–160 ppm benzene–toluene mixture. While the model sufficiently captures the breakthrough onset time and final concentrations of benzene and toluene, it overestimates the maximum outlet concentration of benzene by less than 30% relative error. For toluene, the model predicts a steeper curve than the experiment suggests, thus affecting the prediction of breakthrough onset and completion times. For ε_b_ = 0.38, the breakthrough onset time for benzene is ≈3% later and for toluene ≈9% earlier at the 40–160 ppm inlet concentrations compared to the 100–100 ppm. Breakthrough completion, for the same comparison, comes ≈6% earlier for benzene and ≈30% earlier for toluene. Finally, the breakthrough duration for benzene is ≈16% shorter and for toluene ≈51% shorter at 40–160 ppm inlet concentration, compared to the 100–100 ppm inlet concentration for ε_b_ = 0.38.

Figure 3 introduces the temperature variation in the middle of the column for all scenarios investigated in this work. At first glance, the temperature profiles of the two binaries follow different trends, with acetone–toluene mixtures forming two peaks and the benzene–toluene mixtures forming a single peak. Adsorption is an exothermic process; thus, temperature peaks are expected and signify that adsorption takes place at a specific part of the column. Due to the trace VOC concentrations examined in this work (up to 200 ppm), the magnitude of the temperature rise is below 1 K. Each concentration pair is examined for four potential bed porosity fractions and their corresponding bed lengths. For the acetone–toluene mixture, increasing bed porosity (and thus bed length) leads to the observation of a decreasing maximum temperature, while interestingly, for the benzene–toluene mixtures, the opposite is observed; increasing bed porosity leads to an increasing maximum temperature in the middle of the column. The higher *T*_max_ of the benzene–toluene mixtures compared to acetone–toluene is attributed to the higher heat of adsorption of benzene compared to acetone, as indicated in Table 2.

Figure 4 presents the pressure drop profiles for all of the binary mixtures considered in this paper. Pressure drops follow a linear profile and, in accordance with Ergun’s equation, are equal for corresponding concentrations and bed porosity fractions between the acetone–toluene and benzene–toluene mixtures. The overall values are low, as expected from the column length and flow conditions. The largest pressure drop, 54.86 Pa, is observed for the smallest bed porosity (ε_b_ = 0.38), which then decreases to 30.38 Pa for the largest bed porosity examined (ε_b_ = 0.45) regardless of the mixture composition and component concentration.

## 3 Theoretical Performance Analysis: Hodographs

Multicomponent adsorption equilibrium theory allows the qualitative prediction of the equilibrium behavior of isothermal, Langmuir isotherm-obeying systems. Glueckauf’s work connected chromatographic theory and kinematic waves in a similar manner, which resulted in the ability to predict multicomponent mixture dynamic behavior. The foundation of the theory is the concept of coherence, which assumes that a front of multicomponent fluid is traveling along the column as a front of constant composition and can transition either as a continuous wave or a shock. For example, the feeding of an initially clean bed with a ternary mixture (two low-concentration adsorbable species with an inert carrier gas) generates a front traveling as a shock along the column, whose behavior is critical during industrial operation.

In such a ternary mixture, the equilibrium of each adsorbable component depends on both components, which also, due to the coherence of waves, gives rise to the fundamental quadratic eq 34
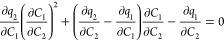
34

Glueckauf’s approach (1949)^40^ enables the binary Langmuir isotherm analysis simplification by the introduction of the *p*_1_ and *p*_2_ variables, where component 2 is assumed to be strongly adsorbed. Therefore, *b*_2_ is always larger than *b*_1_ and eq 34 becomes
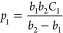
35
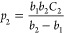
36
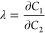
37
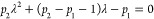
38

Equation 38 has two roots; *M* is the positive and *N* is the negative. The transition of a system from the bed’s initial to the final (feed concentration) state can be described qualitatively on a hodograph plot of *p*_1_ vs *p*_2_. This plot contains two points corresponding to the initial and final state of the bed, respectively, as well as four straight lines passing through them, which correspond to the characteristic curves of the *p*_1_ and *p*_2_ coherence equation’s (eq 38) roots for each point, as follows
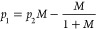
39
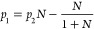
40

Hodograph interpretation relies on 2 rules to predict the system’s dynamic behavior when transitioning from the bed’s initial state to the bed’s final (feed concentration) state^47,48^

1. One departs from the initial composition point on a positive root characteristic line and arrives at the final (feed) composition point on a negative root characteristic curve.
2. Whenever the more strongly adsorbed solute increases in concentration along the column (desorption), we have a diffuse boundary, while where the concentration of the more strongly adsorbed component decreases along the column (adsorption), we have a shock transition.

This shock transition is illustrated in the hodograph plot and allows the prediction of the maximum concentration of the weakly adsorbing component at the column outlet.

### 3.1 Results and Discussion: Hodograph Case Studies

The two simple rules of Hodograph Theory enable the prediction of overshoot concentration in binary mixture dynamic adsorption. To this end, the adsorption of mixtures of acetone with toluene and benzene with toluene (both with air as the inert carrier gas) was studied at three different concentration pairs (160–40, 100–100, 40–160 ppm), each on a clean bed and at *T* = 293.15 K.

**Figure 5:**
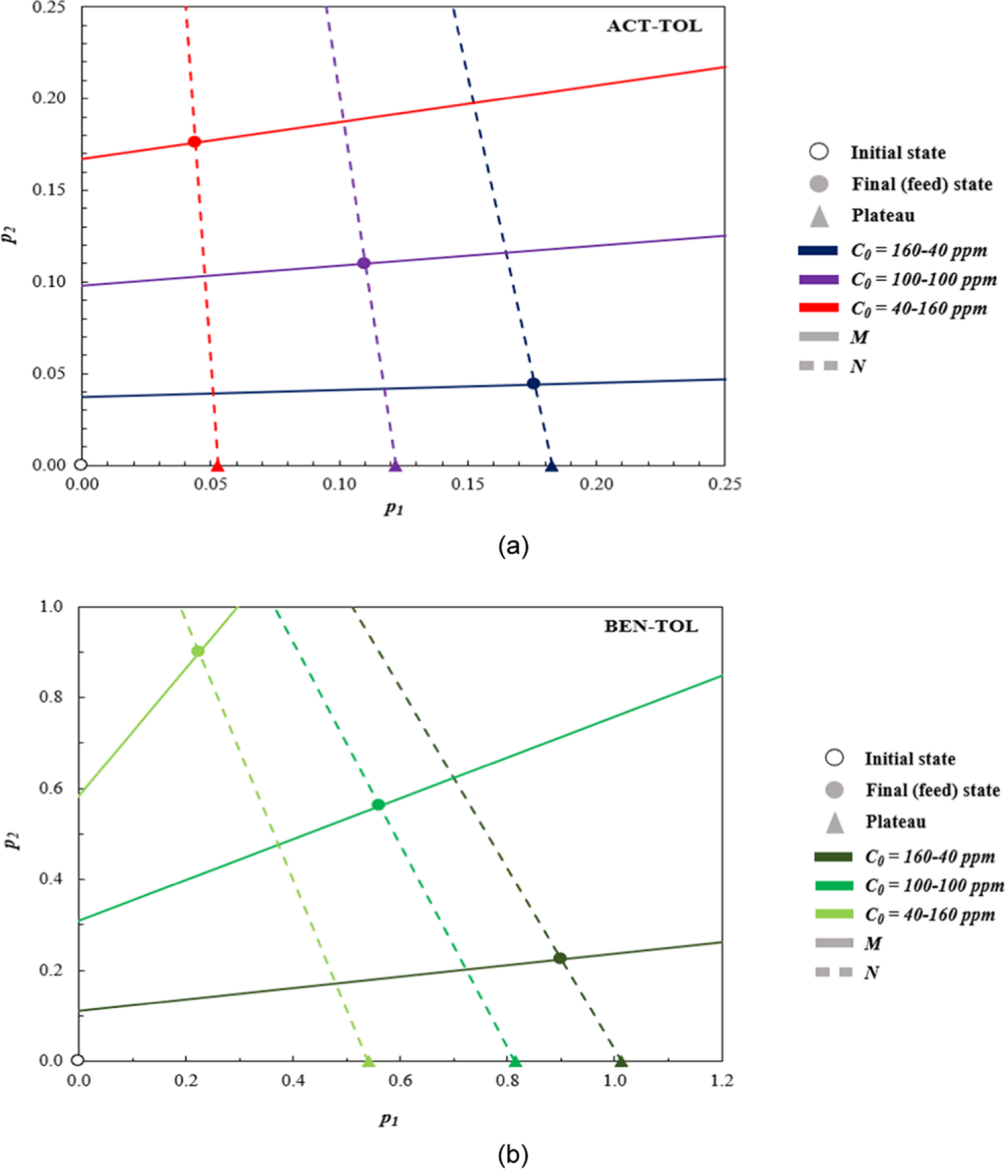
Hodograph plots and theoretical breakthrough predictions for binary mixtures: (a) acetone–toluene (ACT–TOL) and (b) benzene–toluene (BEN–TOL).

For each mixture and inlet concentration pair, the coherence (eq 38) is solved twice. Once for the initial state of the bed and the second for the final (feed) state. For the clean bed scenario, *p*_1_ and *p*_2_ are zero; hence, the *p*_1_ and *p*_2_ axes are considered as the initial state solutions. Only one pair of *M* and *N* values are therefore computed, and thus, 2 curves, corresponding to the final (feed) state roots of the coherence equation, are drawn on the clean bed hodographs (Figure 5).

Table 10 shows the results of the coherence equation solution for the binary mixtures.

**Table 10:** Coherence Equation Solutions for Binary Mixture Hodograph Plot Construction

System	*T* (K)	*C*_0_ (ppm_v_)	State	*p*_1_	*p*_2_	*M*	*N*	Figure 5
ACT–TOL (CLEAN BED)	293.15	160	initial	0	0			a
		40	feed	0.175927	0.043982	25.89115	–0.15449	
	293.15	100	initial	0	0			a
		100	feed	0.109955	0.109955	9.203321	–0.10866	
	293.15	40	initial	0	0			a
		160	feed	0.043982	0.175927	4.984322	–0.05016	
BEN–TOL (CLEAN BED)	293.15	160	initial	0	0			b
		40	feed	0.899281	0.224820	7.951076	–0.50308	
	293.15	100	initial	0	0			b
		100	feed	0.562050	0.562050	2.228027	–0.44883	
	293.15	40	initial	0	0			b
		160	feed	0.224820	0.899281	0.712753	–0.35075	

The clean bed hodographs for the mixtures of acetone–toluene and benzene–toluene at three concentration pairs (160–40, 100–100, and 40–160 ppm) are found in Figure 5. Hodograph plots only rely on pure component Langmuir Isotherm parameters for their construction; thus, each plot is agnostic to bed porosities, lengths, and flow conditions. The predictive capabilities of the hodograph theory can be displayed by referring to Figure 5a. As Rule 1 states, one departs from the initial point on a positive characteristic (*M*) line; therefore, we depart from the initial point on the *p*_1_ axis and arrive at the final (feed) point on the dashed line, which corresponds to the negative characteristic (*N*) line. Hence, if the triangle point is the point where the positive root characteristic (*p*_1_ axis) line and the negative root characteristic line (dashed line) intersect, the route could be summarized as heading to the final (feed) point from the initial point via the triangle point. Since the concentration of the strongly adsorbed component is decreasing due to an amount of it being adsorbed throughout the column, a shock transition occurs, as set out by Rule 2, and thus there is a sharp rise in the concentration, namely, the plateau, of the weakly adsorbed component (acetone/benzene), which exits the column in a higher concentration compared to its inlet.

The hodograph plots of all binary mixtures can be interpreted in an analogous manner. It is interesting to note that the estimated plateau point (triangle) can predict the maximum concentration of the weakly adsorbed component in the column outlet (overshoot). Table 11 summarizes and compares the plateau concentrations derived as predictions from hodograph theory (H), from the experimental data of (E),^5^ and from the simulations (S) in this study. Relative errors are estimated based on eqs 41–43.
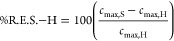
41
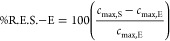
42
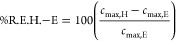
43

**Table 11:** Overshoot Concentration Predictions of Displaced Components from Binary Mixtures

Component	*C*_0_ (ppm_v_)	ε_b_	*C*_max,H_ (mol m^–3^)	*C*_max,E_ (mol m^–3^)	*C*_max,S_ (mol m^–3^)	%R.E.S–H	%R.E.S–E	%R.E.H–E
ACT	160	0.38	0.006909	0.007786	0.007055	2.119	–9.392	–11.272
	160	0.40	0.006909	0.007786	0.007056	2.133	–9.380	–11.272
	160	0.42	0.006909	0.007786	0.007058	2.167	–9.350	–11.272
	160	0.45	0.006909	0.007786	0.007060	2.191	–9.328	–11.272
	100	0.38	0.004609	0.005213	0.004891	6.107	–6.192	–11.592
	100	0.40	0.004609	0.005213	0.004892	6.141	–6.163	–11.592
	100	0.42	0.004609	0.005213	0.004895	6.210	–6.101	–11.592
	100	0.45	0.004609	0.005213	0.004898	6.263	–6.055	–11.592
	40	0.38	0.001997	0.002931	0.002222	11.311	–24.183	–31.887
	40	0.40	0.001997	0.002931	0.002224	11.405	–24.119	–31.887
	40	0.42	0.001997	0.002931	0.002228	11.588	–23.994	–31.887
	40	0.45	0.001997	0.002931	0.002231	11.750	–23.884	–31.887
BEN	160	0.38	0.007488	0.007732	0.007484	–0.062	–3.212	–3.152
	160	0.40	0.007488	0.007732	0.007500	0.151	–3.006	–3.152
	160	0.42	0.007488	0.007732	0.007530	0.557	–2.613	–3.152
	160	0.45	0.007488	0.007732	0.007561	0.965	–2.217	–3.152
	100	0.38	0.006023	0.006598	0.005957	–1.103	–9.720	–8.713
	100	0.40	0.006023	0.006598	0.005995	–0.473	–9.145	–8.713
	100	0.42	0.006023	0.006598	0.006072	0.802	–7.981	–8.713
	100	0.45	0.006023	0.006598	0.006151	2.126	–6.772	–8.713
	40	0.38	0.003996	0.002864	0.003349	–16.191	16.949	39.542
	40	0.40	0.003996	0.002864	0.003399	–14.952	18.678	39.542
	40	0.42	0.003996	0.002864	0.003511	–12.128	22.619	39.542
	40	0.45	0.003996	0.002864	0.003641	–8.896	27.128	39.542

As can be seen in Table 11, the maximum concentration at the column outlet for the weakly adsorbing component (acetone or benzene) slightly increases for the different bed porosities of the simulations carried out in the present paper.

For the binary mixture of acetone–toluene, with acetone at *C*_0_ = 160 ppm, the relative errors between simulation and hodograph theory prediction are ≈2%, between simulation and experiment ≈9%, and between hodograph theory prediction and experiment ≈−11%. When acetone is fed in the column at *C*_0_ = 100 ppm in a mixture with toluene, the relative errors between simulation and hodograph theory prediction are ≈6%, between simulation and experiment ≈−6%, and between hodograph theory prediction and experiment ≈−11%. Finally, for the lowest acetone inlet concentration of 40 ppm in a mixture with toluene, the relative errors between simulation and hodograph theory prediction are ≈11%, between simulation and experiment ≈−24%, and between hodograph theory prediction and experiment ≈−32%.

For the mixture of benzene–toluene, containing benzene at *C*_0_ = 160 ppm, the relative errors between simulation and hodograph theory prediction are <1%, between simulation and experiment ≈−3%, and between hodograph theory prediction and experiment also ≈−3%. For the same mixture, but with components at *C*_0_ = 100 ppm, the relative errors for the overshoot concentration of benzene between simulation and hodograph theory prediction are <2%, between simulation and experiment <−9%, and between hodograph theory prediction and experiment also ≈−9%. Lastly, for the benzene–toluene mixture containing benzene at *C*_0_ = 40 ppm, the relative errors for the overshoot concentration of benzene between simulation and hodograph theory prediction are <−16%, between simulation and experiment <27%, and between hodograph theory prediction and experiment also ≈40%.

From the conducted comparisons, it appears that the developed model can successfully capture component exit concentration overshoot in competitive adsorption. Interestingly, the largest mismatch among all relative error metrics is encountered in the case of a low concentration (40 ppm) of acetone or benzene. There, the largest discrepancies are encountered between the hodograph theory prediction and the published experimental data,^5^ possibly because of the foundations of hodograph theory, which rely on the Langmuir parameters of the pure components for the predictions. The simulation results demonstrate a better agreement with experimental data even on the 40 ppm inlet concentration cases compared to hodograph theory predictions.

## 5 Conclusions

Limiting VOC emissions is of paramount importance in achieving climate protection goals globally, as they pose risks to human health and the environment alike. Pharmaceutical companies, reliant on solvents for the manufacturing of essential active pharmaceutical ingredients, are targeting VOC emissions. Since solvent substitution is not always possible or feasible due to the recipes themselves or stringent regulatory approval processes, emission control technologies such as adsorption are in place to ensure minimal environmental impact. However, the complex and varying waste stream composition reduces the efficiency of adsorption columns, thus increasing the overall process cost.

Although a multitude of scientific studies, both experimental and computational, address adsorption, there is a profound mismatch compared to the proportion of research targeted toward pharma-relevant, multicomponent VOC mixture adsorption.^4,49^ The value of this research, especially under industrially relevant conditions, is profound due to its unique ability to inform and assist in operational decision-making, where experiments are not always possible due to the increased cost and finite resource availability.

The present paper continues to demonstrate the application of a validated, multicomponent, nonisothermal dynamic adsorption model^4^ for simulation and comparison vs published experimental data.^5^ The mixtures of acetone–toluene and benzene–toluene with air as the inert carrier gas are studied under the same three inlet concentration pairs of 160–40, 100–100, and 40–160 ppm at *T* = 293.15 K. First, an estimation of the column length is performed for four different potential bed porosities (ε_b_ = 0.38, 0.40, 0.42, and 0.45) which results in column lengths of *L* = 0.019–0.021 m. Based on these calculations, key breakthrough metrics highlight trends observed across all three mixtures concerning column exit behavior. Specifically, acetone and benzene emerge at the column at a higher concentration compared to their inlet across all concentration pairs examined, thus confirming them as the weakly adsorbed components. The earliest breakthrough onset times occur for acetone when it is in a mixture with toluene. Toluene demonstrates earlier breakthrough onset when in a mixture with benzene compared to the acetone mixtures. As expected, breakthrough onset times decrease with increasing inlet concentration for all three components. The discrepancies between experimental data and simulation results are possibly due to incomplete knowledge of the experimental system details as well as our model limitations. The discrepancies between experimental breakthrough data and simulation results are attributed to the incomplete knowledge of the experimental system details as well as our model limitations.

Moreover, simulation results shed light on temperature variations and pressure drops of multicomponent mixtures. Specifically, due to the trace concentrations of the feed streams studied in this work, the temperature rises in the middle of the column, occurring due to adsorption exothermicity, are minute and slightly higher for the benzene–toluene mixtures compared to the acetone–toluene owing to the higher heat of adsorption for benzene as opposed to acetone. Pressure drop, computed via Ergun’s equation, in the columns examined is minimal, ranging from 30.38 to 54.86 Pa among all mixtures. Higher values are reported for the smallest bed porosity (0.38) and corresponding bed length (0.019 m), which decline as the bed porosity and corresponding calculated bed length increase. These results emphasize the importance of reliable dynamic modeling with respect to adsorption column efficiency and unlock the potential for process optimization on the industrial scale.

Lastly, this paper presents the application of hodograph theory to demonstrate the prediction capability of this simple, fast, and yet first-principles consistent concept on weakly adsorbing component outlet maximum concentration prediction. Specifically, hodograph theory estimations have been compared to our gPROMS dynamic simulation results, showing that the former (albeit originally derived for single-component, isothermal conditions) can provide quick, useful estimates to inform industrial operation before committing resources in pursuit of the latter. However, discrepancies between hodograph theory predictions and experimental data are higher in all cases examined compared with our gPROMS simulation results. Undoubtedly, this paper paves the way for VOC emission control scheduling based on the sequence and duration of solvent breakthrough onset to ultimately optimize activated carbon bed operation and management, not only batch operations but also future continuous pharmaceutical manufacturing.^50^

## Nomenclature

*b*_*i*_: Langmuir affinity coefficient (m^3^ mol^–1^)
*b*_o,*i*_: pre-exponential Langmuir constant (m^3^ mol^–1^)
*C*: component gas-phase VOC concentration (mol m^–3^)
*C*_0,*i*_: inlet concentration of *i* (mol m^–3^)
*C*_max,H_: maximum concentration at column outlet predicted by hodograph theory (mol m^–3^)
*C*_max,S_: maximum concentration at column outlet predicted by simulation (mol m^–3^)
*C*_max,E_: maximum concentration at column outlet obtained by publ. experiment (mol m^–3^)
*C*_pg_: specific heat capacity of gas (J kg^–1^ K^–1^)
*C*_pp_: specific heat capacity of particle (J kg^–1^ K^–1^)
*C*_s0,*i*_: adsorbed phase concentration at equilibrium with *C*_0,*i*_ (mol m^–3^)
*C*_t_: total gas-phase VOC concentration (mol m^–3^)
*D*: bed inner diameter (m)
*D*_AB,*i*_: molecular diffusivity (m^2^ s^–1^)
*D*_eff,*i*_: effective diffusivity of *i* (m^2^ s^–1^)
*D*_k,*i*_: Knudsen diffusivity (m^2^ s^–1^)
*d*_lm_: mean logarithmic column diameter (−)
*d*_p_: particle diameter (m)
*D*_z,*i*_: axial dispersion coefficient (m^2^ s^–1^)
*h*_int_: internal heat transfer coefficient (W m^–2^ K^–1^)
*h*_o_: overall heat transfer coefficient (W m^–2^ K^–1^)
*k*_eff_: effective thermal conductivity (W m^–1^ K^–1^)
*k*_ew_: effective wall thermal conductivity (W m^–1^ K^–1^)
*k*_ez_: effective axial thermal conductivity (W m^–1^ K^–1^)
*k*_f,*i*_: effective mass transfer coefficient of component *i* (m s^–1^)
*k*_g_: gas thermal conductivity (W m^–1^ K^–1^)
*k*_LDF,*i*_: LDF mass transfer coefficient (s^–1^)
*k*_p_: particle thermal conductivity (W m^–1^ K^–1^)
*k*_w_: wall thermal conductivity (W m^–1^ K^–1^)
*L*: bed length (m)
*m*_AC_: mass of activated carbon (kg)
*M*_r_: molecular weight (g mol^–1^)
*P*: pressure (atm only in eq 4)/(Pa)
*q*_*i*_: adsorbed phase VOC concentration (mol m^–3^)
*q*_e,*i*_: equilibrium adsorption capacity of *i* (mol kg^–1^)
*q*_ρe,*i*_: equilibrium adsorption capacity of *i* (mol m^–3^)
*q*_m,*i*_: maximum adsorption capacity of material for component i (mol kg^–1^)
*R*: column inner radius (m)
Re_p_: Reynolds number (adsorbent particle)
*r*_p_: average pore radius (0.99 × 10^–9^ m)
*R*_p_: particle radius (m)
SA: Surface area of adsorbent material (m^2^ g^–1^)
Sc_*i*_: Schmidt number of *i*
Sh: Sherwood number (−)
*T*: temperature (K)
*T*_in_: inlet temperature (K)
*T*_max_: maximum temperature (K)
*T*_w_: wall temperature (K)
*t*_5%,*i*_: breakthrough onset time of component *i* (s)
*t*_95%,*i*_: breakthrough completion time of strongly adsorbing component *i* (s)
*t*_105%,*i*_: breakthrough completion time of weakly adsorbing component *i* (s)
*t*_drt,*i*_: duration of breakthrough (s)
*u*: interstitial velocity (m s^–1^)
*V*_pore_: adsorbent pore volume (6.1 × 10^–4^ m^3^ kg^–1^)
*V*_s_: superficial velocity (m s^–1^)
*x*: wall thickness (m)
α_*0*_: empirical mass diffusion correction factor (20)
Δ*H*_ad,*i*_: heat of adsorption (J mol^–1^)
ε_b_: bulk bed porosity (−)
ε_p_: particle porosity (−)
μ: gas viscosity (Pa s)
ρ_b_: bed density (kg m^–3^)
ρ_g_: gas density (kg m^–3^)
ρ_p_: particle density (kg m^–3^)
*∑ν*: atomic diffusion volume (A: VOC, B: carrier)
τ_p_: particle tortuosity (−)
